# Basal-Like Phenotype in a Breast Carcinoma Case Series from Sudan: Prevalence and Clinical/Pathological Correlations

**DOI:** 10.4061/2011/806831

**Published:** 2011-01-09

**Authors:** Khalid Dafaallah Awadelkarim, Carmelo Arizzi, Elgizouli Omer Musa Elamin, Hussein M. A. Hamad, Pasquale De Blasio, Salwa O. Mekki, Ihsan Osman, Ida Biunno, Nasr Eldin Elwali, Massimo Costanzo Barberis, Renato Mariani-Costantini

**Affiliations:** ^1^Department of Molecular Biology, National Cancer Institute (NCI-UG), University of Gezira, P. O. Box 20, Hospital Street, Wad Medani, Sudan; ^2^Servizio di Anatomia Patologica, Azienda Ospedaliera di Circolo di Melegnano, Via Pandina 1, 20070 Vizzolo Predabissi, Milan, Italy; ^3^Departments of Histopathology & Cytopathology and Oncology, Radiation & Isotope Centre Khartoum (RICK), Algaser Street, P. O. Box 846, Khartoum, Sudan; ^4^Integrated Systems Engineering Srl., Via Fantoli 16/15, 20138 Milan, Italy; ^5^BioRep, Via Fantoli 16/15, 20138, Milan, Italy; ^6^National Health Laboratory, Federal Ministry of Health, P. O. Box 287, Khartoum, Sudan; ^7^Institute for Biomedical Technologies, National Research Council, Via Fratelli Cervi, 93, 20090 Segrate, Milan, Italy; ^8^Department of Basic Sciences, College of Medicine, Al Imam Mohamed Bin Saud Islamic University, P.O. Box 5701, Riyadh 11432, Saudi Arabia; ^9^Department of Pathology, European Institute of Oncology, Via Ripamonti 435, 20141, Milan, Italy; ^10^Department of Oncology and Experimental Medicine, “G. d'Annunzio” University and Unit of Molecular Pathology and Genomics, Aging Research Center (CeSI), “G. d'Annunzio” University Foundation, Via Colle dell'Ara, 66013 Chieti, Italy

## Abstract

Basal-like breast cancer, an aggressive subtype associated with high grade, poor prognosis, and younger age, is reported frequently in Africa. We analyzed the expression of the basal cytokeratins (CKs) 5/6 and 17 in a case series from Central Sudan and investigated correlations among basal CK status, ER, PgR, and Her-2/neu, and individual/clinicopathological data. Of 113 primary breast cancers 26 (23%), 38 (34%), and 46 (41%) were, respectively, positive for CK5/6, CK17, and combined basal CKs (CK5/6 and/or CK17). Combined basal CK+ status was associated with higher grade (*P* < .03) and inversely correlated with ER (*P* < .002), PgR (*P* = .004) and combined ER and/or PgR (*P* < .0002). Two clusters based on all tested markers were generated by hierarchical cluster analysis and k-mean clustering: I: designated “hormone receptors positive/luminal-like” and II: designated “hormone receptors negative”, including both basal-like and Her-2/neu+ tumors. The most important factors for dataset variance were ER status, followed by PgR, CK17, and CK5/6 statuses. Overall basal CKs were expressed in a fraction of cases comparable to that reported for East and West African case series. Lack of associations with age and tumor size may represent a special feature of basal-like breast cancer in Sudan.

## 1. Introduction

Cytokeratins (CKs) are used as differentiation markers in breast cancer (BC), since their expression is thought to remain stable in carcinogenesis [1]. In breast ducts CK8 and CK18 are expressed in the luminal layer whereas CK5/6, CK14, and CK17 characterize the basal layer [2–4]. Thus BC may be luminal or basal with regard to CK phenotype, with some tumors coexpressing both basal and luminal CKs [2]. This is supported by microarray expression profiling that classifies BC into five prognostically and clinically relevant molecular subtypes, luminal A, luminal B, basal-like, Her-2/neu, and normal breast-like [5–16]. Accordingly, BC can no longer be viewed as a single biologic and pathologic entity, which implies a need for stratified rather than unified approaches for research, prevention, and treatment [17].

The basal-like subtype overlaps, but is not synonymous, with the triple negative subset, which includes BCs that do not express ER, PgR, and Her-2/neu and tend to occur at a younger age and in patients with pathogenetic *BRCA1* mutations [18–21]. Approximately 85% of the ER–/Her-2/neu– BCs are of basal-like phenotype [9]. Most importantly, although most basal-like BCs do not express ER, PgR, or Her-2/neu, in case series of different origin 14% to 45% of the cases were reported to express at least one of these markers [7, 9, 14].

Basal-like/triple negative BCs initially respond to chemotherapy in the neoadjuvant setting, but their overall prognosis remains poor [14]. Importantly, the tumors with worst prognosis seem to be those expressing basal CKs [5, 7, 8, 22] or epidermal growth factor receptor (EGFR) [9, 23].

Basal-like BCs show common as well as heterogeneous morphologic, genetic, and immunophenotypic features, and, up to date, there is no international consensus regarding their exact definition [5–12, 20]. Basal CKs, which have been shown to be independently associated with poor outcome [7, 9, 24–26], are expressed in most, but not all, BCs classified as basal-like by immunohistochemical (IHC) or gene microarray analysis [3, 7, 20, 27–29]. Furthermore in a subset of BCs basal CKs are coexpressed with other markers, including EGFR, P-cadherin, c-KIT, caveolin 1, and p63, although consideration of such markers does not appear to improve the identification of the cases with poor outcome compared to basal CKs alone [20]. Therefore Rakha et al. [20] suggested to rely on basal CK expression alone to define basal-like BC, remarking that, in spite of shared clinicopathologic and IHC features, basal CK-positive BCs and basal-like BCs are not strictly the same entity [7, 29].

Genetic, ethnic, and racial factors influence BC phenotypes, possibly by determining intrinsic differences in tumor biology [6, 30, 31]. In this regard, it is remarkable that basal-like/triple negative BC appears to be more common in African American women [6, 12, 32] and in BC case series from West and East Africa (range: 22%–34%), where it seems to be also associated with features indicative of poor prognosis [33–36].

In a previous study we found that a BC case series from Khartoum, Central Sudan, was comparable to one from Milan, Northern Italy, in combined hormone receptors status and BC subtypes [37]. Relative to the Italian patients, the Sudanese patients were younger and their tumors were larger, of higher grade and more advanced in stage [37].

We address here the question of the BC subtypes identified by clustering analyses within the Sudanese BC case series. To this end, we re-evaluated, using more sophisticated statistical analyses, the expression of the basal CKs 5/6 (CK5/6) and 17 (CK17) in relation to estrogen/progesterone receptors (ER/PgR), human epidermal growth factor receptor 2 (Her-2/neu), and the available clinicopathological and individual data. We refer in this paper to two designations of BCs with basal subtype: (i) basal CK+, defining BCs that express basal CKs regardless of the expression of other markers [20] and (ii) basal-like, identified by the triple-negative CK-positive profile (ER−/PgR−/Her-2/neu−/basal CK+).

## 2. Materials and Methods

### 2.1. Patients

The study is based on a series of 113 Sudanese cases of primary invasive BC diagnosed between 2004-2005 at the Department of Histopathology & Cytopathology of the Radiation and Isotope Center Khartoum (RICK), Khartoum, Sudan. This series, retrospectively selected to include all consecutively accessioned BCs with available paraffin-embedded material adequate for immunohistochemistry (as determined by immunostaining with control antibodies), was previously used to compare pathological, clinical, and prognostic characteristics of BC in Sudan versus Italy [37]. Exclusion criteria were as follows: (a) in situ carcinomas, (b) sarcomas, and (c) secondary tumors. Overall, the most frequent histotype was invasive ductal carcinoma, which accounted for 101/113 cases (89.4%). Other histotypes were invasive lobular (5/113, 4.4%), mucinous (5/113, 4.4%), medullary (1/113, 0.9%), and Paget's (1/113, 0.9%). Some of the included invasive ductal carcinomas were also associated with other features: (i) inflammatory invasive ductal carcinoma (1/113), (ii) lactating adenoma associated with invasive ductal carcinoma (1/113), (iii) invasive ductal carcinoma with squamoid differentiation (1/113), and (iv) invasive ductal carcinoma showing features of pleomorphic carcinoma with cartilaginous differentiation (1/113). Histological grading was performed using the Nottingham Combined Histologic Grade (NCHG) system [38]. The breast tumors included in this study were of intermediate grade (grade 2: 35/113; 31%) and high grade (grade 3: 78/113; 69%). The intermediate-grade tumors included all the mucinous carcinomas (5/5, 100%), 3 of the 5 lobular carcinomas (3/5, 60%), and 27/101 (26.7%) of the invasive ductal carcinomas. On the other hand, the high-grade tumors included the unique cases of Paget's and medullary carcinomas and the remaining invasive ductal carcinomas (74/101, 73.3%). 

Age and tumor size were recorded only in 73 and 88 of the 113 cases, respectively. Most patients presented with advanced disease and were lost to followup, as it frequently occurs in developing countries [39–41]. Lack of data on lymph node status and follow up precluded correlations with stage and prognosis [37]. According to data from the Sudan Federal Ministry of Health, 78% of the Sudanese BC patients have stage III or IV disease [42, 43]. 

### 2.2. Immunohistochemistry

Whole consecutive sections were immunostained for ER (clone 1D5, Dako), PgR (clone PgR 636, Dako), Her-2/neu (polyclonal, Dako), CK5/6 (clone D5/16 B4, Dako), CK17 (clone E3, Dako) and, as quality controls of antigenic preservation, for the CK pool (clones AE1–AE3, Dako) and vimentin (clone V9, Dako). IHC results were recorded as percentages of immunostained cells in ≥2000 neoplastic cells. Only nuclear reactivity was taken into account for ER and PR, which were classified as negative, when absent or present in <5% of the neoplastic cells, or positive, when present in ≥5% of the neoplastic cells. Only intense and complete cell membrane immunoreactivity in ≥10% of the cells was taken as evidence of Her-2/neu overexpression (score 3+) [44]. Borderline Her2/neu cases (score 2+) were reassessed by fluorescence in situ hybridization (FISH), as previously described [37]. Basal CKs 5/6 and 17 were regarded as positive when any cytoplasmic and/or cell membrane staining was seen [6, 9, 37].

## 3. Statistical Analyses

Unsupervised hierarchical cluster analysis (CA) was done for hormone receptors (ER, PgR), Her-2/neu and basal CK (CK5/6 and/or CK17) statuses to determine the natural clustering of the BCs according to the studied IHC markers. CA was performed using squared Euclidean distance measurements to obtain a dissimilarity matrix. Ward's method was then applied to this matrix to build a tree [45]. This method uses analysis of variance to evaluate distances between clusters, minimizing the sum of squares of any two hypothetical clusters that can be formed at each step. CA was done using SPSS statistical package version 15.0 (SPSS Inc., Chicago, IL).

Unsupervised *k*-mean clustering algorithm, performed with STATISTICA 7.0 (StatSoft, Inc., Tulsa, Ok), was applied to confirm and explore better the generated cluster(s). The *k*-mean clustering used the Euclidean distance as the similarity metric [46].

Data reduction was done by factor analysis, applying principal components analysis (PCA) to the selected variables (ER, PgR, Her-2/neu, CK5/6, and CK17) to determine the minimum number of factors, among those considered, that retained most of the dataset variance, and to quantify the significance of the explained variance for each variable in dataset grouping(s). A scoring algorithm, that loaded each individual variable most strongly onto the factor with which it was most correlated, created summary factors. The adopted extraction methods were the Kaiser criterion, that is, the sum of squared factor loadings (eigenvalue) >1 [47] and the scree test, that identifies the cut-off discriminating important from unimportant factors in the plot of the eigenvalues [48]. A default setting of 25 maximum iterations of algorithm steps to obtain convergence was used to extract factors. Factor scores were shown graphically. Statistical analyses were developed by SPSS statistical package version 15.0 (SPSS Inc., Chicago, IL). Factor score loadings were interpreted by rule of thumb in confirmatory factor analysis as follows: ≥0.7: higher factor; <0.7–≥0.6: high factor; <0.6–≥0.4: central factor; <0.4–≥0.25: low factor; <0.25: lower factors [49, 50]. Higher factors build on the rationale that the 0.7 level corresponds to about half of the variance in the indicator being explained by the factor. However, being the 0.7 standard high for real-life data, for exploratory purposes lower levels were used, down to 0.7, with 0.4 for the central factor and 0.25 for other factors [49, 50].

All cut-off values were determined before the statistical procedures. Correlations between different variables were calculated using *χ* ^2^ test or *t*-test. Significance was set at <.05. All *P* values were two-tailed.

## 4. Results

### 4.1. Immunohistochemical Characteristics and Basal Cytokeratin Status

Table 1 summarizes the basal cytokeratin status in the studied case series. Of 113 primary BCs 26 (23%), 38 (34%), 18 (16%), and 46 (41%) were respectively positive for CK5/6, for CK17, for CK5/6 and CK17, and for CK5/6 and/or CK17. The frequency of the basal CK+ subtype (basal CKs+ regardless of other markers) was therefore 46/113 (41%), whereas the basal-like subtype as defined by triple-negative CK+ profile (ER−/PgR−/Her-2/neu−/basal CK+) was 11/113 (10%). Moreover, the frequency of basal-like subtype as synonymous of triple negative, regardless of CK status, was 18/113 (15.9%) (Table 2). Combined positive basal CK status (CK5/6+ and/or CK17+) was associated with higher grade (*P* < .03, Table 3) and was inversely correlated with the expression of ER and PgR (resp., *r* = −0.3, *P* < .002; *r* = −0.27, *P* = .004, Table 3). A highly significant negative correlation emerged when combined hormone receptor status (ER+ and/or PgR+) was considered (*r* = −0.36, *P* < .0002, Table 3).

There was no association between basal CK status and Her-2/neu (Table 3). However, as basal CK+ status, Her-2/neu+ status was inversely correlated with the expression of ER and PgR (resp., *r* = −0.27, *P* = .004; *r* = −0.26, *P* = .005), and with combined ER+ and/or PgR+ status (*r* = −0.28, *P* = .003). Basal CK status was not associated with age at diagnosis (available for 73 cases) and tumor size (available for 88 cases) (Tables 3 and 4); however, although not significant, the mean age of the patients with basal CK+ tumors was lower compared to that of the patients with basal CK− tumors (49.8 ± 15.8 years versus 51.2 ± 14.1 years, Table 4), and the mean tumor size was smaller (4.5 ± 2.7 cm versus 5.4 ± 3.4 cm, Table 4). All the lobular (5/113) and mucinous tumors (5/113) were ER+/PgR+/Her-2/neu− (luminal type) and all were negative for the basal CKs, except one mucinous tumor that was found to be positive for CK5/6. The unique cases of Paget's (1/113) and medullary (1/113) carcinomas were both found to be ER−/PgR−/Her-2/neu+/basal CK−(Her-2/neu subtype). 

Therefore, the tumors positive for the basal CKs were invasive duct carcinomas (98%), except a single mucinous carcinoma (Table 3). No association emerged between basal CKs expression and BC histotype (Table 3).

### 4.2. Cluster Distribution and Factor Analysis

**Figure 1:**
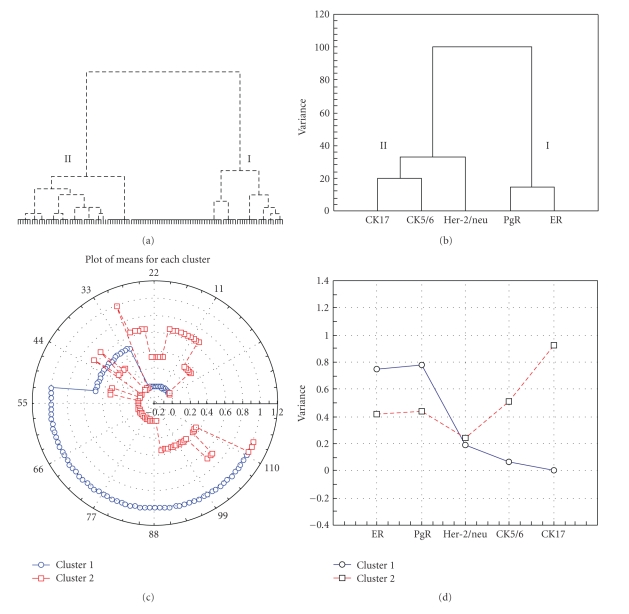
Two clusters generated based on the statuses of basal cytokeratins (CK5/6, CK17), hormone receptors (ER, PgR), and Her-2/neu by hierarchical cluster analysis ((a) & (b)) and k-mean clustering ((c) & (d)). Cases in each cluster are shown in (a) and (c). The factor(s) that contribute to each cluster are shown in (b) and (d).

Two major clusters of patients were generated using hierarchical cluster analysis (Figure 1(a)): cluster I with 65/113 (57.5%) patients and cluster II with 48/113 (42.5%) patients. Clustering the five tested IHC markers revealed that hormone receptors (ER, PgR) clustered in I whereas the basal CKs (CK 5/6, CK 17, and Her-2/neu clustered in II, each in a separated branch (Figure 1(b)). Hence, cluster I could be designated as “hormone receptors positive/luminal-like,” whereas cluster II as “hormone receptors negative,” including both the basal-like and the Her-2/neu+ subtypes [5, 22, 26].

Comparable results were obtained through *k*-mean clustering, with 72/113 (63.7%) patients joining cluster I and 41/113 (36.3%) cluster II (Figure 1(c)). In addition *k*-mean clustering revealed that hormone receptors (ER, PgR) and basal CKs (CK 5/6, CK 17) played a major role in identifying clusters I and II, respectively (Figure 1(d)). On the other hand, Her-2/neu played quite similar roles in the determination of the two clusters, with slightly higher weight in cluster II (Figure 1(d)).

**Figure 2:**
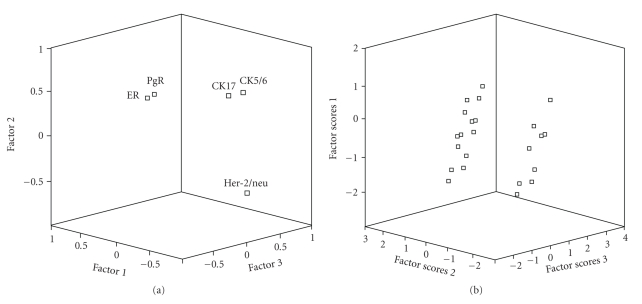
(a) Score components of three factors extracted for the tested dataset variables. Factor analysis showed that three factors explained 80.3% of the dataset variance. The first factor extracted (eigenvalue = 2.1) accounted for the largest proportion of variance (42.3%) and corresponded to hormone receptor status (with loads of ER: 0.80 and PgR: 0.78). The second factor (eigenvalue = 1.2) explained 23.4% of variance and corresponded to basal cytokeratins status (with loads of CK17: 0.55 and CK5/6: 0.54). The third factor (eigenvalue = 0.7, with a load of 0.6 for Her-2/neu status), a factor that explained 14.6% of the variance. (b) Individual factor scores of the three of the five extracted factors. Note that some samples were superimposed. Factor scores were extracted by regression method.

**Figure 3:**
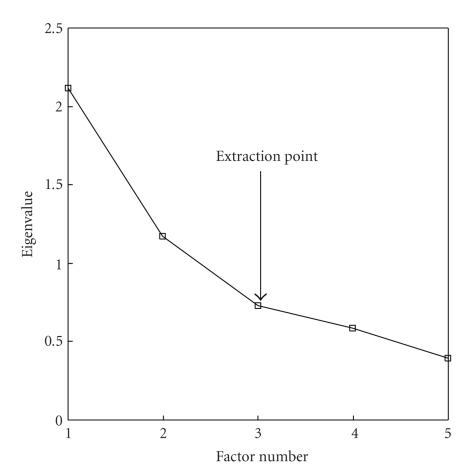
Scree plot of the eigenvalues. The adopted extraction methods were the Kaiser criterion, that is, the sum of squared factor loadings (eigenvalue) >1, and the scree test, that is, the place where the smooth decrease of eigenvalues appears to level off to the right of the plot of the eigenvalues.

Factor analysis showed that three factors explained 80.3% of the dataset variance (Figures 2(a) and 2(b)). The first factor (eigenvalue = 2.1) accounted for the largest proportion of variance (42.3%) and corresponded to hormone receptor status (loads: ER: 0.80; PgR: 0.78), while basal CKs (loads: CK17: −0.6: CK5/6, −0.59) and Her-2/neu (load: −0.39) statuses were negatively loaded on this factor. The second factor (eigenvalue = 1.2) explained 23.4% of variance and corresponded to basal CK status (loads: CK17, 0.55; CK5/6, 0.54), while Her-2/neu status (load: −0.68) loaded negatively on this factor. The third factor, corresponding to Her-2/neu status (eigenvalue = 0.7, with a load of 0.6), explained 14.6% of the variance (Figure 2(a)). Individual factor scores of the extracted factors are shown in Figure 2(b). Other two factors needed to be extracted to explain the complete dataset variance, that is, factor 4, corresponding to CK17 status (eigenvalue = 0.6, load: 0.56), that explained 11.7% of the variance, while CK5/6 (load: −0.49) loaded negatively on this factor and factor 5, corresponding to ER status (eigenvalue = 0.4, load: 0.45), that explained 8% of the variance, while PgR (load: −0.43) loaded negatively on this factor. The Scree plot of the eigenvalues is shown in Figure 3. The component matrix of these five factors is shown in Table 5. Of note, these analyses are in support of the proposal of Rakha et al. [20] who suggested to rely on basal CK expression alone (basal CK+ subtype) to define basal-like BC, regardless of the status of the other markers. In fact, our analyses assigned all the BCs that expressed basal CKs, regardless of the other markers, to cluster II. Furthermore, the basal-like subtype (BCs with triple-negative phenotype that express basal CKs: ER−/PgR−/Her-2/meu−/basal CKs+) was also included in cluster II. It is worth mentioning that the adoption of the latter criterion only for the definition of basal BC would miss many cases, as the basal-like subset accounted for only 10% of the cases versus 41% for the basal CKs+ subset.

## 5. Discussion

The expression of basal CKs is a negative prognostic marker, implying resistance to therapy and poor prognosis, particularly in the context of BCs with triple-negative status [12, 25, 26, 35, 51]. Basal-like BC, which largely overlaps with triple-negative BC, is a well-recognized BC subtype with the above-mentioned clinically-relevant implications [12, 25, 26, 35, 51]. Basal-like/triple-negative BC appears to occur more frequently in African American women and in breast cancer case series from East and West Africa, which could reflect intrinsic differences in tumor biology related to racial/ethnic factors [6, 12, 21, 30, 32].

A better understanding of the impact of basal-like/triple negative BC in BC series from native African women would contribute to the assessment of the influence of race on this particularly relevant BC subtype. It is important to develop BC prevention and treatment policies in African populations, that, with increased life expectancy, are predicted to face marked increases in BC rates [12, 14, 28, 35, 52, 53].

Recent studies found that the basal-like phenotype was frequent in West (Nigeria and Senegal) and East (Uganda) African BC case series (range: 22% to 27%), where it was also associated with features of poor prognosis [33–36]. In contrast, we [37] and Adebamowo et al. [54] reported lower frequencies of basal-like BC subtype (as defined by triple-negative, basal CK+ phenotype) in Sudanese (10%) and Nigerian BC series (15.8%), which was mainly due to the markedly higher frequency of hormone receptor positivity found in these tumor series (Sudan: ER: 64%; PgR: 67%; ER and/or ER: 75%, Nigeria: ER+: 65.1%; PgR: 54.7%), as compared to the other studies from Africa [33–37, 54].

Consideration of two basal subtypes, that is, basal CK+, defined by expression of basal CKs regardless of other markers [20], and basal-like, defined by the triple-negative CK+ phenotype (ER−/PgR−/Her-2/neu−/basal CK+), may explain these discrepancies. In fact, in our BC series from Central Sudan, the frequency of basal-like BC is 10%, as previously reported [37], but that of basal CK+ BC is 41%. This reflects the presence of an excess of cases that express basal CKs together with ER/PgR and/or Her-2/neu.

In the present Sudanese BC series the frequency of basal CK+ status (41%) appears to be much higher than those reported for Western Caucasian and also for African American BC series (13–20% and 26%, resp.), but results quite comparable to the 34% frequency found in a BC series from Kyadondo County in Uganda and to the 33% frequency reported from West Africa (Nigeria and Senegal) [20, 25, 29, 33, 35, 51]. In the study of Adebamowo et al., basal CKs were not investigated and the basal-like subtype was defined by triple-negative phenotype only (ER−, PR−, and Her-2/neu−) as one category [54]. In this regard it is notable that the Nigerian and the Sudanese case series yield almost the same frequencies of basal-like BCs defined by triple negative phenotype only: 15.8%, that is, 24/152, in the Nigerian series and 15.9%, that is, 18/113, in the Sudanese series [37, 54].

In our Sudanese series, basal CK expression was associated with higher histologic grade and with hormone receptor negative status. This is in agreement with well-established evidence that the expression of basal markers occurs in poorly differentiated hormone receptors-negative BCs, as reported for Caucasian and African American series and also for the Ugandan series [25, 26, 35, 51, 55]. As in other studies, CK17 was more frequently positive than CK5/6 [25].

It is well established that in both African-American and Caucasian BC series the expression of basal CKs is significantly related to younger age at BC onset [26]. In our Sudanese series basal CK status was not associated with age at disease diagnosis, as also reported for the series from Kyadondo County in Uganda [35]. However, although not significant, the mean age and the mean tumor size were lower in the basal CK+ group than in the basal CK− one. The lack of significance for the difference in age may be due to the fact that the patients were mostly young, reflecting the young age at disease diagnosis typical of the institutional BC series from the Sudan [37, 56–58].

Indeed, the higher frequency of basal-like phenotype in African case series could be partially explained by the younger age of the patients [33–36]. However, socioeconomic, genetic, ethnic, and lifestyle/reproductive factors are also likely to be involved [30, 37]. In particular, emerging data reported that certain reproductive factors (i.e., extended breast-feeding/lactation, high parity, and early menarche) may have a greater impact on risk of certain molecular BC subtypes compared to others [59, 60]. Furthermore, other confounding factors, like antigen degradation of archival formalin fixed, paraffin-embedded tissue blocks, should also be considered for the reportedly high frequency of hormone receptor negativity, with subsequently higher frequencies of both basal-like BC identified by the triple-negative CK+ profile (ER−/PgR−/Her-2/neu−/basal CK+) and unclassified triple-negative types [33, 36, 37, 54, 61].

The lack of association between basal CK+ status and larger tumor size is quite unexpected [51]. This unusual finding might reflect the fact that large size at presentation, due to late disease diagnosis, is one of the main features of BC in Sudanese patients, when compared to BC in patients from Europe and North America [9, 37, 62, 63]. Due to longer survival, this could result in a relative enrichment of less aggressive subtypes among the BCs of larger size [37, 64], a hypothesis that requires to be further investigated in larger and prognostically well-characterized BC series from Sudan.

Except one mucinous carcinoma, all the basal CK+ tumors were invasive duct carcinomas, consistent with the literature data [51]. The fact that all the invasive lobular tumors were basal CK− could be relevant but could also reflect a bias due to the relatively low frequency of this histotype in the study series and needs further evaluation on a larger number of cases.

In concordance with the gene expression-based IHC subtypes defined in Western BC case series [5, 22, 26], clustering based on the five tested IHC markers outlined a hormone receptors-positive/luminal-like cluster and a hormone receptors-negative cluster with basal CKs (CK5/6, CK17) and Her-2/neu. As expected, factor analysis showed that hormone receptor status was the factor that most influenced dataset variance among the other tested factors, being negatively affected by both basal CK and Her-2/neu statuses. Basal CK status was in second position, with Her-2/neu status loaded negatively on this factor, although this was not supported by a direct negative correlation. Her-2/neu status was in the third place. The other two extracted factors (factor 4: CK17 status, and factor 5: ER status) had minimum effects as extracted factors on the dataset variance. Collectively, this demonstrates that the most important factors in the dataset were ER status, followed by PgR, CK17, and CK5/6 statuses.

Her-2/neu status played a complex role in the dataset variance, as it negatively affected both hormone receptor status (which was consistent with statistical correlations) and basal CK status (as demonstrated only by factor analysis). As previously reported, the basal-like phenotype and the Her-2/neu expression are inversely correlated [9, 14, 65, 66], and it is likely that the nonbasal-like tumors include a high prevalence of Her-2/neu amplified tumors [65]. In this regard, it should be considered that the effects of Her-2/neu on the determination of the two clusters were quite similar, being only slightly in favour of cluster II (Figure 1(d)). Interestingly, Harris et al. reported that the expression of basal markers was strongly associated with Her-2/neu+ BCs not responding to preoperative therapy based on trastuzumab plus vinorelbine [53]. This underlines the need to better verify the BC subsets in which basal CKs, Her-2/neu and hormone receptors could interact, in African and non-African case series.

## 6. Conclusion

In the presently studied BC series from Central Sudan the frequency of the tumors expressing basal CKs was much higher than the frequencies reported for Caucasian and African-American BC series, but it was comparable to that found in BC series from East and West Africa [20, 25, 29, 33, 35, 51]. This suggests that the impact of the tumors expressing basal CKs could be higher in sub-Saharan African patients, a possibility that needs to be confirmed by additional studies in different African populations. In Sudan a higher impact of the tumors expressing basal CKs could be ascribed to a variety of factors, including racial/genetic factors, environmental and reproductive factors, population structure, and sampling/referral bias. However, while an early age of onset is one of the clinical characteristics associated with BC expressing basal CKs, in our case series basal CK-positive status was associated with higher grade and hormone receptor-negative status, but not with age at disease diagnosis and tumor size. This quite unexpected lack of association might reflect a selective effect of late disease diagnosis. The most important factors for clusterization in distinct BC subsets were ER status, followed by PgR, CK17, and CK5/6 statuses. As in West Africa, the identified clusters were in concordance with the gene expression-based immunohistochemical subtypes defined in Western BC case series [5, 22, 26, 33], despite the difference in patient population. However, the overall frequency of basal-like subtype (ER−/PgR−/Her-2/neu−/basal CK+) was low (10%, in Sudanese; 15.8%, in Nigerian), which was mainly due to the reported markedly higher frequency of hormone receptor positivity (ER: 64%; PgR: 67%; ER and/or ER: 75% in Sudanese and ER+: 65.1%; PgR: 54.7% in Nigerian) as compared to the other studies from Africa [33–37, 54].

## Figures and Tables

**Table 1:** Basal cytokeratins in the studied case series.

	Number (%)
CK5/6	
Positive	26 (23)
Negative	87 (77)
CK17	
Positive	38 (34)
Negative	75 (66)

Combined (CK5/6 and/or CK17)	
Positive	46 (41)
Negative	67 (59)

**Table 2:** Basal breast cancer frequencies in the currently studied case series, according to different designations.

BC basal subtype	Designation	Frequency
Basal CK+	basal CKs+ regardless of the expression of other markers (basal CK+)	46/113 (41%)
Basal-like/triple- negative	triple-negative (ER−/PgR−/Her-2/neu−)	18/113 (15.9%)
Basal-like	triple-negative CK-positive profile (ER−/PgR−/Her-2/neu−/basal CK+)	11/113 (10%)

**Table 3:** Basal cytokeratins status according to tumor grade, tumor size (*T*), ER, PgR, combined ER/PgR, Her-2/neu, and histology.

CK5/6+ and/or CK17+, Number (%)			
	Positive	Negative	*χ* ^2^
Grade			
g2	9 (20)	26 (39)	4.72 (*P* < .03)
g3	37 (80)	41 (61)	
Tumor size (*T*)			
*T*1	4 (9)	6 (9)	0.67 (*P* = .88)
*T*2	21 (45.5)	25 (37)	
*T*3	7 (15)	13 (19.5)	
*T*4	5 (11)	7 (10.5)	
NA^¶^	9 (19.5)	16 (24)	
ER			
ER+	21 (46)	50 (75)	9.8 (*P* < .002)
ER−	25 (54)	17 (25)	
PgR			
PgR+	23 (50)	51 (76)	8.2 (*P* = .004)
PgR−	23 (50)	16 (24)	
Combined ER/PgR			
ER+ and/or PgR+	25 (54)	58 (87)	14.5 (*P* < .0002)
ER−/PgR−	21 (46)	9 (13)	
Her-2/neu			
Her-2/neu+	10 (22)	14 (21)	0.012 (*P* = .9)
Her-2/neu−	36 (78)	53 (79)	
Histology			
IDC*	45 (98)	56 (84%)	6.3 (*P* = .17)
ILC°	—	5 (7%)	
Mucinous	1 (2)	4 (6%)	
Medullary	—	1 (1.5%)	
Paget's disease	—	1 (1.5%)	

^¶^NA: not available tumor size data in 25 cases, *IDC: infiltrating ductal carcinoma, °ILC: infiltrating lobular carcinoma.

**Table 4:** Basal cytokeratins status according to patient's age at disease diagnosis and to tumor size.

CK5/6 and/or CK17			
	Positive	Negative	*t*-test
Age (years)*			
Mean ± SD^¶^	49.8 ± 15.8	51.2 ± 14.1	(*t* = 0.57; *P* = .57; 95% CI −5.6–9.08)
Range	25–80	30–70	
Mean tumor size (cm)^#^			
Mean ± SD	4.5 ± 2.7	5.4 ± 3.4	(*t* = 0.58; *P* = .56; 95% CI −0.85 –1.55)
Range	1–15	1–14	

*The mean age of this series was 51.2 ± 14.3 years (range: 25–80 years), age was missing for 40 cases. ^¶^SD: standard deviation; ^#^the mean tumor size of this series was 4.7 ± 2.8 cm (range: 1–15 cm), size was missing for 25 cases.

**Table 5:** Component matrix of the five factors extracted by principal component analysis (PCA).

	Factor 1	Factor 2	Factor 3	Factor 4	Factor 5
ER	0.804	0.228	0.288	0.134	0.448
PgR	0.784	0.252	0.366	−0.015	−0.433
Her-2/neu	−0.391	−0.678	0.612	0.112	0.012
CK5/6	−0.585	0.538	0.351	−0.488	0.080
CK17	−0.598	0.553	0.123	0.564	−0.051
